# Cortical Correlates of Visuospatial Switching Processes Between Egocentric and Allocentric Frames of Reference: A fNIRS Study

**DOI:** 10.1007/s10548-023-01032-0

**Published:** 2024-02-05

**Authors:** Renato Orti, Yann Coello, Francesco Ruotolo, Marion Vincent, Angela Bartolo, Tina Iachini, Gennaro Ruggiero

**Affiliations:** 1 Laboratory of Cognitive Science and Immersive Virtual Reality, CS-IVR, Department of Psychology, University of Campania L. Vanvitelli, Viale Ellittico, 31, 81100 Caserta, Italy; 2 UMR 9193, SCALab, Sciences Cognitives et Sciences Affectives, Université de Lille, 59000 Lille, France

**Keywords:** Egocentric-allocentric reference frames, Visuospatial switching/non-switching processes, fNIRS, Frontal areas, Temporo-parietal junction, Dorsal and ventral attention networks

## Abstract

Human beings represent spatial information according to egocentric (body-to-object) and allocentric (object-to-object) frames of reference. In everyday life, we constantly switch from one frame of reference to another in order to react effectively to the specific needs of the environment and task demands. However, to the best of our knowledge, no study to date has investigated the cortical activity of switching and non-switching processes between egocentric and allocentric spatial encodings. To this aim, a custom-designed visuo-spatial memory task was administered and the cortical activities underlying switching vs non-switching spatial processes were investigated. Changes in concentrations of oxygenated and deoxygenated haemoglobin were measured using functional near-infrared spectroscopy (fNIRS). Participants were asked to memorize triads of geometric objects and then make two consecutive judgments about the same triad. In the non-switching condition, both spatial judgments considered the same frame of reference: only egocentric or only allocentric. In the switching condition, if the first judgment was egocentric, the second one was allocentric (or vice versa). The results showed a generalized activation of the frontal regions during the switching compared to the non-switching condition. Additionally, increased cortical activity was found in the temporo-parietal junction during the switching condition compared to the non-switching condition. Overall, these results illustrate the cortical activity underlying the processing of switching between body position and environmental stimuli, showing an important role of the temporo-parietal junction and frontal regions in the preparation and switching between egocentric and allocentric reference frames.

## Introduction

Long-standing research in the field of spatial cognition has shown that spatial information can be represented in memory according to an egocentric (body-centred) and an allocentric (object-centred) frame of reference (e.g., O’keefe and Nadel 1979; Paillard 1991; McNamara 2002; Burgess 2006; Avraamides and Kelly 2008). Neurofunctional evidences have shown bilateral activations, more right sided, of the fronto-parietal network for egocentric encodings (e.g., Committeri et al. 2004; Zaehle et al. 2007), and activations, more left sided, of the posteromedial and medio-temporal substructures for allocentric encodings (Vallar et al 1999; Galati et al. 2000; Committeri et al. 2004; Parslow et al. 2004; Zaehle et al. 2007; Antonova et al. 2009; Schindler and Bartels 2013; Chen et al. 2014; Ruotolo et al. 2019; Derbie et al. 2021a; Moraresku et al. 2023).

Previous cognitive models suggested that spatial memory is mainly supported by egocentric representations, with allocentric representations serving for reorientation processes (Wang and Spelke, 2002; see also Cheng and Newcombe, 2005; Gallistel, 1990). However, in the light of the revision of the Wang and Spelke’s model, Waller and Hodgson (2006) argued that egocentric and allocentric spatial representations cooperate. Indeed, in our daily activities we rely on both spatial representations to respond to specific environmental needs and task demands. For example, when we use a fork, we encode its position relative to our body and relative to other objects in space, i.e., the plate. This example illustrates the cooperation (i.e., “switching”) between the egocentric and allocentric spatial reference frames that is required to cope with the natural complexity of the surrounding environment (Nadel and Hardt 2004; Burgess 2006; Harris et al. 2012; Ruggiero et al. 2018a, b; Orti et al. 2023).

So far, few behavioral studies have provided evidence about the switching processes between egocentric and allocentric reference systems either in spatial-navigation or visuo-spatial memory tasks (Harris and Wolbers 2014; Harris et al. 2012; Morganti and Riva 2014; Ruggiero et al. 2018a; see also Ruggiero et al. 2018b). For example, Harris and colleagues (Harris et al. 2012; Harris and Wolbers 2014) compared the performance of healthy elderly participants with that of young adults on a spatial navigation task in virtual environments aimed to assess the switching processes between egocentric- and allocentric-based spatial strategies. The results suggested an age-related decline of spatial switching processes: elderly participants performed worse than young adults when required to switch between spatial strategies. Similarly, Morganti et al. (2013) have found a selective impairment of spatial switching processes in patients diagnosed with Alzheimer’s disease (AD), especially when switching from an allocentric to an egocentric spatial strategy was required. More recently, Ruggiero et al. (2018a) measured the ability to switch between egocentric and allocentric spatial representations in amnestic Mild Cognitive Impairment (aMCI) and AD patients compared to healthy controls, using of an ad-hoc devised visuo-spatial memory task. The results showed that aMCI and AD patients performed worse than healthy controls in switching from allocentric to egocentric spatial representations. In addition, AD patients were also impaired in switching from egocentric to allocentric spatial representations. Overall, these results are consistent with the progressive deterioration, in typical and pathological ageing, of the brain structures supposed to underly the switching processes between reference frames. Specifically, the visuo-spatial switching processes are thought to be mediated by the activity of the locus coeruleus noradrenaline system (LCNA) and prefrontal cortex (PFC) (Aston-Jones, and Cohen 2005; Harris et al. 2012; Morganti et al. 2013; Pai and Yang 2013), together with some posteromedial brain structures (Maguire 2001; Byrne et al 2007; Vann et al. 2009; Wolbers and Hegarty 2010; Ruggiero et al. 2014, 2018a; Boccia et al. 2017; Mitchell et al. 2018).

In this regard, Burgess and colleagues (Bicanski and Burgess 2020; Bird et al. 2012; Byrne et al. 2007; see also; Burgess 2006, 2008; Evans et al. 2016) proposed a model of spatial memory that aims to explain how egocentric and allocentric frames of reference combine. The authors speculate that body-centered spatial representations mediated by posterior parietal regions and object- or environment-centred spatial representations mediated by medio-temporal regions are translated by posteromedial structures such as the retrosplenial cortex (RSC) (for a recent review of the role of RSC in the translational process between reference frames see Vann et al. 2009; Alexander et al. 2023).

However, the encoding of reference frames likely also involves visuo-spatial attentional processes, as we need to dynamically select and use cues from the external environment while ignoring others. In this regard, dorsal and ventral attentional networks are expected to play important roles in egocentric and allocentric spatial encoding, respectively (Corbetta and Shulman 2002; Corbetta et al. 2008; Vossel et al. 2014), and presumably in the switching processes between them.

To date, while attempts have been made to investigate the cortical correlates of non-visuospatial switching tasks (see Cutini et al. 2008; Laguë-Beauvais et al 2013; Vasta et al. 2018), to the best of our knowledge no study has yet explored the cortical correlates underlying the translation processes between spatial reference frames. To fill this gap, in the present study the functional near-infrared spectroscopy (fNIRS) neuroimaging technique was used. Using a virtual version of the Ego-Allo Switching Task (Ruggiero et al. 2018a, b; Orti et al. 2023), participants had to perform switching (from egocentric-to-allocentric: Ego-Allo; from allocentric to egocentric: Allo-Ego) and non-switching (only egocentric: Ego-Ego; only allocentric: Allo-Allo) spatial judgments about relative distances between memorised triads of geometric objects (e.g., sphere, cube). For each triad, participants were asked either two questions about the object’s location using the same reference frame (i.e., non-switching condition), or two questions about the object’s location using two different reference frames (i.e., switching condition). This experimental paradigm was based on previous studies with healthy adults (Iachini and Ruggiero 2006; Ruggiero et al. 2016, 2021), neurological patients (Ruggiero et al. 2014, 2018a, 2020), blind people (Iachini et al. 2014; Ruggiero et al. 2018b), and has demonstrated its effectiveness in discriminating between spatial reference frames.

As for fNIRS, it aims to non-invasively map haemodynamic responses induced by neural activity by measuring changes in oxygenated (HbO) and deoxygenated (HbR) haemoglobin relative concentrations (Boas et al. 2014; see Pinti et al. 2019 for a review), thus complementing fMRI. Among the strengths of this neuroimaging technique are its cost-effectiveness, portability, and the possibility of using it with experimental paradigms not suited for fMRI. In addition, fNIRS experimental setups allow participants to remain upright, unlike fMRI experimental setups where participants lie supine. This is particularly important in experimental paradigms where participants have to encode spatial information, as preserving the natural upright position allows for experimental settings with higher ecological validity.

Based on previous literature (Galati et al. 2000; Committeri et al. 2004; Ruotolo et al. 2019), we expected that the direct comparison between egocentric- and allocentric-based spatial judgments would reveal the involvement of the fronto-parietal areas in egocentric-based processes in both switching and non-switching conditions, more right sided. In addition, we also expected the involvement of the frontal areas along with some temporo-parietal regions in allocentric- vs egocentric-based information in both switching and non-switching conditions. Finally, if transient body-centered spatial representations stored in the fronto-parietal regions are translated into stable object-centred spatial representations stored in medio-temporal regions and vice versa, then the concurrent activation of the fronto-parieto-temporal regions in switching compared to non-switching conditions is expected to emerge (Burgess 2006, 2008).

## Method

### Participants

The appropriate sample size for the study was determined by means of an a priori power analysis using G*Power, version 3.1.9.4 (Faul et al. 2009) with the following parameters: Cohen’s effect size d = 0.50 (between medium and large, van Elk et al. 2009), α = 0.05, Power (1 − β) = 0.90. The minimum total sample size was 36.

Forty-two participants aged between 18–35 years were recruited for the study. Four participants did not undergo to the fNIRS registration due to technical problems with the equipment. The final sample consisted of 38 participants (27 females), aged between 18 and 35 years of age (M_age_ = 22.86, SD_age_ = 4.08; M_education_ = 15.35, SD_education_ = 3.6). All participants had normal or corrected-to-normal vision, no reported motor, sensory, neurological, or psychiatric disorders, and were all right-handed as assessed by the Edinburgh Handedness Inventory (Oldfield 1971) (EHI score > 0.5). Each participant gave informed consent to participate in the study. Participants were recruited and tested in accordance with the requirements of the relevant local ethics committee (ethics approval number 2021-482-S92, April 2021) and the 2013 Declaration of Helsinki.

### Stimuli

**Fig. 1:**
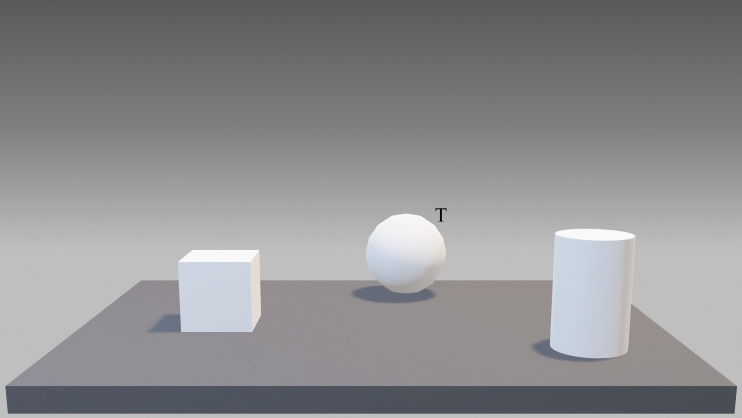
Example of stimuli. The figure illustrates the triad of objects (i.e., cube, cylinder, sphere) placed on a grey panel. “T” represents a target object (e.g., the sphere), which is the reference point used to make the allocentric judgement (see Iachini et al. 2014)

A virtual version of the Ego-Allo Switching Task (Iachini and Ruggiero 2006; Ruggiero et al. 2018a, b) was developed by using SketchUp Make (Trimble, USA). Six 3D geometric objects (cone, cylinder, cube, parallelepiped, pyramid, sphere) of large (8 × 8 cm, but parallelepiped and cylinder 8 × 11 cm) and small size (6 × 6 cm, but parallelepiped and cylinder 6 × 9 cm) were designed and arranged in two series (A and B) and presented on twenty-four textured plasterboard panels (each measuring 50 × 30 × 2 cm). The panels were presented centrally in front of the participants. Each triad was arranged according to the following criteria: (i) the distances between the objects were clearly perceived; (ii) the level of metric difficulty in comparing egocentric and allocentric distances was the same for all judgments; (iii) each triad was placed on the table corresponding to the participants’ midsagittal plane. An example of a triad is shown in Fig. 1. In this case, the distances between the stimuli were: cube-sphere = 11 cm, sphere-cylinder = 28 cm, cylinder-cube = 17 cm. The cube and the cylinder were respectively 6 cm and 12 cm from the edge. The cube was the target, i.e., the reference point for the allocentric judgments. The metric difference between the two objects closest to the body (12-6) and the two objects closest to the sphere (17–11) was the same, i.e., 6 cm.

### Setting

The experiment took place in a soundproof room of the research platform IrDIVE (Innovation Research in the Digital and Interactive Visual Environments) of the Research Federation: Visual Sciences and Cultures (FR 2052 SCV, Tourcoing, France). The stimuli were presented centrally in front of the participants on a 24.0″ PC screen (Asus VG248QE, Wide Screen 16:9 FullHD), with a screen resolution of 1920 × 1080 pixel, and a refresh rate of 60 Hz. Participants sat in a chair in front of a desk on which the PC screen and a keyboard were placed, approximately 35 cm from the PC screen. The experiment was set up and run using the E-Prime 2.0 software (v 2.0, Build 2.0.10.356—Psychology Software Tools, USA).

### Procedure

#### Training Phase

Before starting the experiment, participants were given written instructions about the task, which were repeated orally: they were asked to memorise the objects and their relative positions as accurately as possible. During the training phase (5 min), participants familiarised with the entire experimental procedure. At the beginning of each experimental session, the 3D geometric objects used in the experiment were presented one by one, and participants were asked to name them aloud to avoid possible naming problems. The learning phase began as soon as participants reported that they had fully understood the task.

#### Learning Phase

**Fig. 2:**
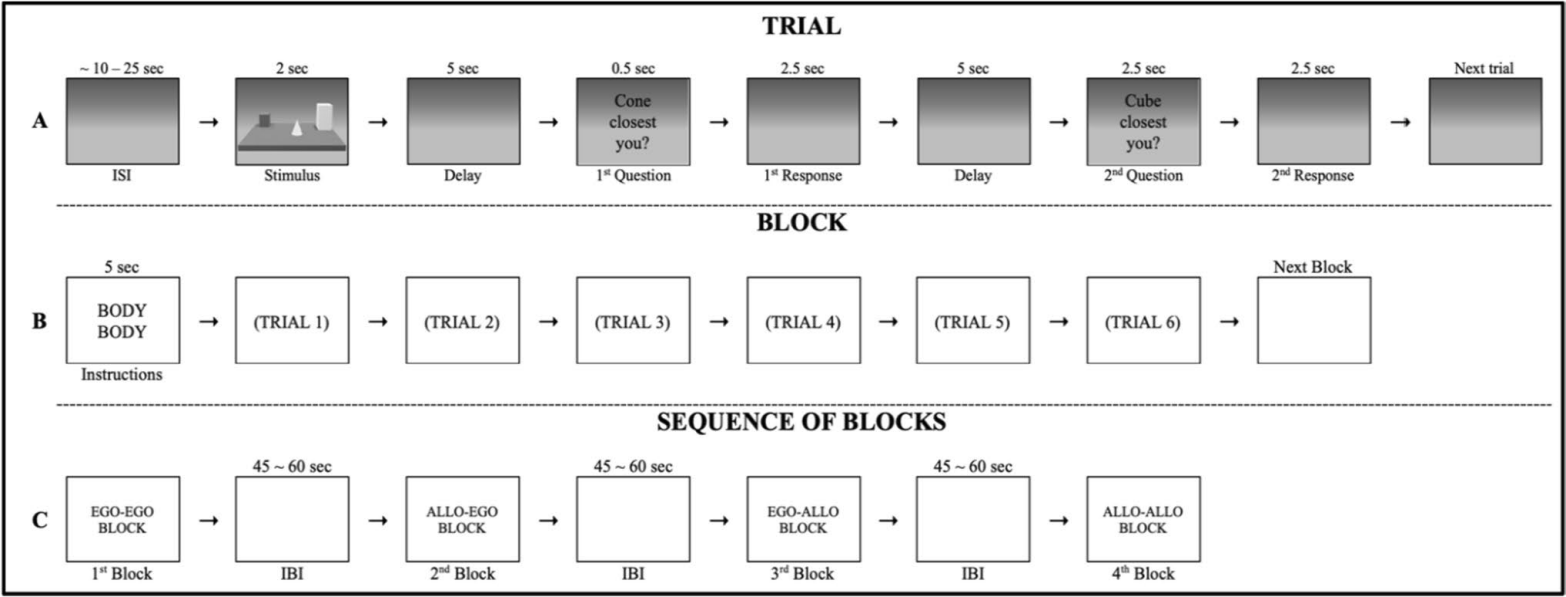
Experimental flow. The figure shows an example of a trial (**A**), a block (**B**) and sequence of blocks (**C**). **A** Each trial started with an ISI (random duration between 10 and 25 s). The stimulus was presented for 2 s followed by a 5 s delay. Two spatial judgments were then required. Each question appeared for 0.5 s, then participants had 2.5 s to make a spatial judgement through a motor response. A second delay of 5 s was presented between the 1st and 2nd spatial judgments. **B** Each block began with a brief instruction, presented for 5 s, informing the participant of the pair of spatial judgments to be made. The following instructions could appear BODY–BODY (two egocentric spatial judgments); OBJECT–OBJECT (two allocentric spatial judgments); BODY–OBJECT (one egocentric and one allocentric spatial judgment); OBJECT–BODY (one allocentric and one egocentric spatial judgment). **C** Each sequence contained the four spatial tasks

Participants were asked to memorise as accurately as possible the triads (objects and their positions) presented for 2 s. Then the triad disappeared, and after a 5-s delay during which a blank was shown, the test phase began (see Fig. 2A for a schematic representation of the experimental procedure).

#### Testing Phase

Participants were asked to make egocentric and allocentric spatial judgments of relative distances via motor responses (key presses). The egocentric questions were of the type: “*Was object X the closest to you?*”; the allocentric questions were of the type “*Was object X the closest to object Y*?”. Both egocentric and allocentric questions were presented in a shorter form (e.g., egocentric: “*X closest you?*”; allocentric: “*X closest Y*?”). The questions appeared in front of the participants for 0.5 s. As soon as the question disappeared, participants gave a motor response within a time window of 2.5 s by pressing the key assigned to “Yes” or “No” (the keys assigned to “Yes” and “No” were counterbalanced across participants).

To assess switching and non-switching judgments for each triad, participants had to make two spatial judgments in succession. For the switching condition, two judgments were required that involved two different anchor points (e.g., from-egocentric-to-allocentric and from-allocentric-to-egocentric), whereas for the non-switching condition, two judgments were required that involved the same frame of reference (e.g., from-egocentric-to-egocentric or from-allocentric-to-allocentric). Twenty-four triads were presented, 12 for the switching conditions and 12 for the non-switching conditions. The 12 switching triads were associated with 24 questions, 12 of which required a switch from an egocentric to an allocentric reference frame (Ego-Allo block) and 12 of which required a switch from an allocentric to an egocentric reference frame (Allo-Ego block). The 12 non-switching triads were associated with 24 questions, 12 of which required two subsequent egocentric (Ego-Ego) spatial judgments and 12 of which required two subsequent allocentric (Allo-Allo) spatial judgments. Triads were associated with questions in counterbalanced order and presented randomly. This controlled for material and order effects.

A block design was used for stimulus presentation and four blocks of six trials each were presented: two switching blocks (Ego-Allo, Allo-Ego), two non-switching blocks (Ego-Ego, Allo-Allo) (see Fig. 2B). In order to homogenise the level of difficulty of the four blocks, each block was preceded by a short 5-s instruction aimed at informing the participant about the pair of spatial judgments he/she had to make (e.g. “BODY–BODY” for two egocentric spatial judgments, i.e. the “Ego-Ego block”; “OBJECT–OBJECT” for two allocentric spatial judgments, i.e. the “Allo-Allo block”; “BODY–OBJECT” for an egocentric and then an allocentric spatial judgment, i.e. the “Ego-Allo block”; “OBJECT–BODY” for an allocentric and then an egocentric spatial judgment, i.e. the “Allo-Ego block”). Each trial started with a jittered inter-stimulus interval (ISI) that randomly varied between 10 and 25 s per trial (Fig. 2B). The blocks were presented in a counterbalanced order across the participants and with a jittered inter-block interval (IBI) that randomly lasted between 45 and 60 s (Fig. 2C).

As this was a single-group study, participants underwent two consecutive fNIRS scan sessions, one for each probe montage (Fronto-Parietal, Occipito-Temporal; see “Probe design” section for further details about probe montages). Thirty minutes of resting state were allowed between the two fNIRS scan sessions.

Overall, accuracy (1 = correct; 0 = incorrect) and response times (in sec) were collected for each judgement (total = 48 judgements) as behavioural measures, while changes in relative concentrations of oxygenated (ΔHbO) and deoxygenated (ΔHbR) haemoglobin in switching (Ego-Allo, Allo-Ego) and non-switching (Ego-Ego, Allo-Allo) conditions were recorded as cortical correlates.

### fNIRS Apparatus, Data Acquisition and Pre-processing

#### Apparatus

The FOIRE-3000 continuous-wave fNIRS system from Shimdzu (Shimadzu Co., Japan), equipped with 32 optodes (16 emitters, 16 receivers), was used to measure the relative concentration changes of oxygenated (ΔHbO) and deoxygenated (ΔHbR) haemoglobin during the experimental sessions. Specifically, the relative concentrations of HbO and HbR were recorded for the entire trial duration from stimulus onset until the end of the second question (i.e., 18 s). The system operated at three different near-infrared wavelengths (780, 805, 830 nm) with a sampling frequency of 4 Hz (i.e. 250 ms temporal resolution).

#### Probes Design

**Fig. 3:**
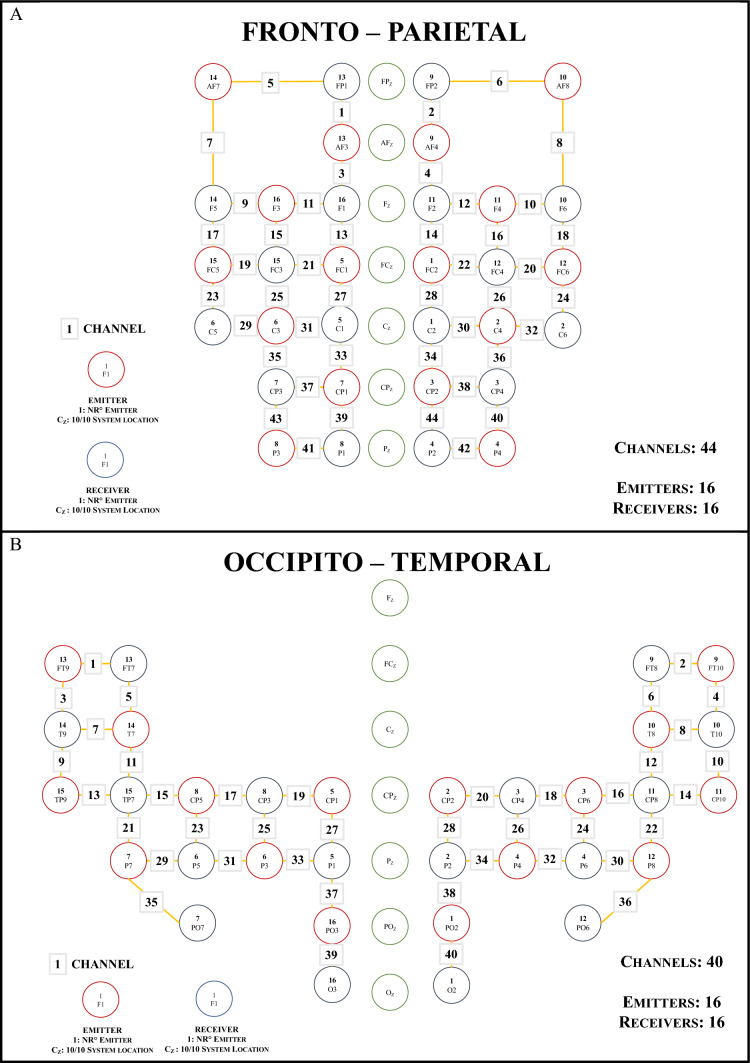
fNIRS probe montages*.* Schematic representation of the **A** fronto-parietal and **B** occipito-temporal fNIRS probe montages. The emitters are shown in red and the receivers in blue. The optodes were placed on the scalp according to the 10/20 system, with the ‘Cz’ (i.e., vertex) as the reference point. The distance between the optodes was fixed at 3 cm. Channels where haemodynamic activity was measured are shown as white numbered squares

Two 16 × 16 optode probes were designed using the Matlab toolboxes FOLD (Zimeo Morais et al. 2018) and AtlasViewer (Aasted et al. 2015) to cover most of the fronto-parietal and parieto-occipitotemporal brain regions bilaterally, respectively. Probe placement was performed according to the international 10/20 system, using ‘Cz’ as a reference point (the midpoint between the nasion and the inion) for each optode probe montage. In Fig. 3A and B, a schematic representation of the two probe montages is shown, with emitters and receivers as circles in light and dark grey, respectively, and channels (in the middle between emitter and receiver) as numbered white squares (odd channels on the left hemisphere, even channels on the right hemisphere). The inter-optic distance between emitter and receiver was fixed at 3 cm. Specifically, the fronto-parietal montage resulted in a 44-channel montage (22 in each hemisphere), whereas the occipitotemporal montage resulted in a 40-channel montage (20 in each hemisphere).

#### fNIRS Data Pre-processing

The pre-processing of the fNIRS data was performed according to the most recent literature on fNIRS guidelines (Pinti et al. 2019; Yücel et al. 2021; see also Ayaz et al. 2022). First, a quality check of the fNIRS signal was performed using the MATLAB toolbox “QT-NIRS” (Hernandez and Pollonini 2020). The quality of fNIRS signals was quantified channel-by-channel in each montage as the cardiac pulsation strength of raw fNIRS signals in the temporal and spectral domains, by combining the scalp coupling index (SCI; Pollonini et al. 2014) and the peak spectral power (PSP; Pollonini et al. 2016), respectively, at all-time points of user-defined time windows and then expressed as a percentage (0–100). Channels below the quality threshold (i.e., 80%) were excluded from further analysis (≤ 5% in fronto-parietal and occipito-temporal montages). The remaining pre-processing steps were performed using the MATLAB toolbox “Homer 3” (Huppert et al. 2006b). Identification and correction of motion artefacts were performed using the wavelet-based filtering algorithm (Molavi and Dumont 2012; Brigadoi et al. 2014). The relative concentration of oxygenated (ΔHbO) and deoxygenated (ΔHbR) haemoglobin was then calculated using the modified Beer-Lambert law (Delpy et al. 1988), and a band-pass filter with a low cut-off at 0.010 Hz and a high cut-off at 0.20 Hz was used to attenuate physiological noise fluctuations in the fNIRS signal. Individual haemodynamic response functions (HRFs) were baseline corrected on a trial-by-trial basis: the relative concentrations of ΔHbO and ΔHbR during the 2 s prior to trial onset were subtracted from the overall haemodynamic activity. The HRF for each experimental condition (Ego-Ego, Allo-Allo, Ego-Allo, Allo-Ego) was then estimated by block-averaging ΔHbO and ΔHbR.

### Statistical Analysis

For the behavioural task, a repeated-measures ANOVA with Spatial Judgments (Ego-Ego, Allo-Allo, Ego-Allo, Allo-Ego) as a four-level within-variable on the average of the first and second spatial judgments was performed on mean accuracy (0–1) and RT (sec), respectively. Each participant’s mean accuracy was calculated as the percentage of correct responses (wrong = 0, correct = 1, range of scores for each condition = 0–12). This was obtained by dividing the total number of correct responses for combined conditions (e.g., Ego-Ego or Allo-Ego) by 12 (i.e., the maximum accuracy). The Tukey test was used to analyse post-hoc effects.

For the neurofunctional data, a series of one-tailed t-tests were performed to compare the mean concentrations of ΔHbO, ΔHbR in each experimental condition with respect to baseline, and to compare the mean concentrations of both chromophores between conditions. Type I errors due to multiple comparisons were controlled by the False Discovery Rate method (Benjamini and Hochberg 1995) with q = 0.05 (hereafter “CorrFDR”).

## Results

### Behavioural Results: Accuracy and Response Times

Descriptive analyses of accuracy and response time for the switching/non-switching conditions are reported in Table 1.

**Table 1:** Mean values and standard deviations of accuracy (0–1) and RT (sec) for non-switching and switching conditions

Visuo-spatial memory tasks							
Accuracy (mean %)				Response time (sec)			
Non-switching		Switching		Non-switching		Switching	
Ego-Ego	Allo-Allo	Ego-Allo	Allo-Ego	Ego-Ego	Allo-Allo	Ego-Allo	Allo-Ego
M (SD)	M (SD)	M (SD)	M (SD)	M (SD)	M (SD)	M (SD)	M (SD)
.83 (.03)	.64 (.03)	.73 (.02)	.71 (.02)	0.64 (.06)	1.07 (.06)	0.89 (.07)	0.97 (.07)

Regarding accuracy, a significant effect of Spatial judgments emerged (F (3, 111) = 17.70, *p* < 0.001, ${\eta }_{p}^{2}$ = 0.324) due to Ego-Ego spatial judgements being more accurate than all other spatial judgements (at least *p* < 0.005), and Ego-Allo spatial judgments being more accurate than Allo-Allo and Allo-Ego spatial judgements (at least *p* < 0.05).

As regards response times, a significant effect of Spatial Judgments was also found (F (3, 111) = 23.79, *p* < 0.001, ${\eta }_{p}^{2}$ = 0.391) with Ego-Ego spatial judgements being faster than all other judgements (*p* < 0.001), and Ego-Allo spatial judgments being faster than Allo-Allo ones (*p* < 0.05).

### Behavioural Results: Correlations

No significant correlations emerged between the mean accuracy and RTs of non-switching (Ego-Ego, Allo-Allo) and switching (Ego-Allo, Allo-Ego) spatial judgments. Therefore, no speed–accuracy trade-off effects were observed.

### fNIRS Results: Spatial Conditions vs Baseline

All comparisons between reference frames and baseline are reported in Table 2.

**Table 2:** Channels with a significant increase in both Hb chromophores (HbO, HbR) during the study compared to baseline

Contrast	Ch	Regions	Right/Left	Hb	*t* value	*p*-value	Cohen’s d
EE > Bas	29	Precentral gyrus	L	HbR	3.34	< 0.01	0.59
	5	Superior frontal gyrus	L	HbR	2.97	< 0.01	0.51
	2	Superior frontal gyrus	R	HbR	2.85	< 0.01	0.50
	8	Middle frontal gyrus	R	HbR	2.51	0.01	0.42
	4	Superior frontal gyrus	R	HbR	2.47	0.01	0.42
AA > Bas	25	Precentral gyrus	L	HbR	2.82	< 0.01	0.49
	7	Middle frontal gyrus	L	HbO	2.80	< 0.01	0.45
	1	Superior frontal gyrus	L	HbO	2.40	0.01	0.39
EA > Bas	7	Middle frontal gyrus	L	HbO	3.77	< 0.01	0.64
	16	Inferior frontal gyrus	R	HbO	2.78	< 0.01	0.46
	18	Inferior frontal gyrus	R	HbO	2.69	0.01	0.45
	42	Superior parietal gyrus	R	HbO	2.58	0.01	0.45
	40	Inferior parietal gyrus	R	HbO	2.44	0.01	0.42
	41	Superior parietal gyrus	L	HbO	2.37	0.01	0.39
	9	Middle frontal gyrus	L	HbO	2.36	0.01	0.40
	5	Superior frontal gyrus	L	HbO	2.27	0.02	0.39
	26	Inferior parietal gyrus	R	HbO	2.11	0.02	0.38
	8	Middle frontal gyrus	R	HbO	2.04	0.02	0.35
	17	Supramarginal gyrus	L	HbO	2.04	0.02	0.34
	20	Inferior frontal gyrus	R	HbO	2.03	0.02	0.33
	14	Middle temporal gyrus	R	HbO	1.79	0.04	0.30
	24	Superior temporal gyrus	R	HbO	1.77	0.04	0.30
AE > Bas	18	Inferior frontal gyrus	R	HbO	3.21	< 0.01	0.55
	7	Middle frontal gyrus	L	HbO	3.06	< 0.01	0.50
	5	Superior frontal gyrus	L	HbO	2.62	0.01	0.44

Only significant results after correction (Corr_FDR_) are reported

*EE* ego-ego, *AA* allo-allo, *EA* ego-allo, *AE* allo-ego, *Bas.* baseline

#### Ego-Ego vs Baseline

The results of the direct comparisons between Ego-Ego spatial judgments with respect to the baseline are reported. A spread increase of ΔHbR was found bilaterally in frontal regions, more precisely in channels 2, 4, 5, 8 and 29 covering rostro-caudally the Superior, Precentral and Middle Frontal gyri (at least *p* < 0.05 Corr_FDR_).

#### Allo-Allo vs Baseline

The results of direct comparisons of Allo-Allo spatial judgments with respect to baseline are reported. Significant increases in both ΔHbO and ΔHbR in the left hemisphere were found in frontal regions, specifically in channels 1, 7, 25 covering rostro-caudally the superior, precentral, and middle frontal gyri (at least *p* < 0.05 CorrFDR).

#### Ego-Allo vs Baseline

The results of the direct comparisons of Ego-Allo spatial judgments with respect to baseline are reported. A diffuse increase of ΔHbO was observed bilaterally in Fronto-Parieto-Temporal regions. Such significant increases of ΔHbO were found in channels 5, 7–9, 14, 16–18, 20, 24, 26, 40–42 covering rostro-caudally the Superior, Middle, and Inferior Frontal gyri, Superior and Inferior Parietal gyri, the Supramarginal gyrus, the Superior and Middle Temporal gyri (at least *p* < 0.05 Corr_FDR_).

#### Allo-Ego vs Baseline

The results of the direct comparisons of Allo-Ego spatial judgments with respect to baseline are reported. An increase of ΔHbO was found bilaterally in frontal regions, more precisely in channels 5, 7 and 18, covering rostro-caudally the Superior, Middle, and Inferior Frontal gyri (at least *p* < 0.05 Corr_FDR_).

### fNIRS Results: Comparisons Between Spatial Conditions

#### Ego-Allo vs Ego-Ego

The results of the direct comparisons between Ego-Allo and Ego-Ego spatial judgments are reported in Table 3. Higher concentrations of both ΔHbO and ΔHbR were found in Ego-Allo spatial judgments compared to Ego-Ego ones. The results showed higher concentrations of ΔHbO bilaterally in frontal and parietal regions, more specifically in channels 3, 7, 9, 16, 23, 41, 44 located above Superior, Middle, and Inferior Frontal gyri, as well as above Superior and Inferior Parietal gyri. Regarding ΔHbR, higher concentrations were found bilaterally in parietal regions, more specifically in channels 18, 20, 32, 39, located above the Superior and Inferior Parietal gyri, Supramarginal and Angular gyri (at least *p* < 0.05 Corr_FDR_).

**Table 3:** Channels with a significant increase in both Hb chromophores (HbO, HbR) between spatial conditions

Contrast	Ch	Regions	Right/Left	Hb	*t* value	*p*-value	Cohen’s d
EA > EE	20	Inferior parietal gyrus	R	HbR	3.65	< 0.01	0.62
	16	Inferior frontal gyrus	R	HbO	3.31	< 0.01	0.56
	9	Middle frontal gyrus	L	HbO	3.25	< 0.01	0.56
	32	Angular gyrus	R	HbR	3.07	< 0.01	0.51
	3	Superior frontal gyrus	R	HbO	2.95	< 0.01	0.50
	41	Superior parietal gyrus	L	HbO	2.92	< 0.01	0.47
	39	Superior parietal gyrus	L	HbR	2.89	< 0.01	0.48
	23	Inferior frontal gyrus	L	HbO	2.59	0.01	0.43
	18	Supramarginal gyrus	R	HbR	2.57	0.01	0.44
	7	Middle frontal gyrus	L	HbO	2.57	0.01	0.43
	44	Inferior parietal gyrus	R	HbO	2.37	0.01	0.39
**EE > EA**	5	Superior parietal gyrus	R	HbR	4.24	< 0.01	0.73
	29	Inferior frontal gyrus	L	HbR	3.81	< 0.01	0.66
	8	Superior frontal gyrus	R	HbR	3.16	< 0.01	0.54
	4	Precentral gyrus	R	HbR	3.00	< 0.01	0.51
	31	Superior frontal gyrus	L	HbR	2.94	< 0.01	0.50
	7	Superior parietal gyrus	L	HbR	2.84	< 0.01	0.46
	16	Precentral gyrus	R	HbR	2.81	< 0.01	0.48
	9	Precentral gyrus	L	HbR	2.55	0.01	0.44
	10	Superior parietal gyrus	R	HbR	2.48	0.01	0.41
	2	Superior frontal gyrus	R	HbR	2.42	0.01	0.42
	27	Inferior frontal gyrus	R	HbR	2.31	0.01	0.43
	23	Precentral gyrus	L	HbR	2.22	0.02	0.37

Only significant results after correction are shown

*EE* ego-ego, *EA* ego-allo

For the Ego-Ego spatial judgments only higher concentrations of ΔHbR were found bilaterally in Fronto-Parietal regions. More specifically, the results showed higher concentrations of ΔHbR in channels 2, 4, 5, 7–9, 10, 16, 23, 27, 29, 31, covering rostro-caudally Superior and Inferior Frontal gyri, Precentral and Superior Parietal gyri (at least *p* < 0.05 Corr_FDR_).

#### Allo-Ego vs Allo-Allo

The results of the direct comparisons between Allo-Ego and Allo-Allo spatial judgments are reported in Table 4. Higher concentrations of ΔHbO were found in channel 21, located above the left Superior Frontal gyrus, for Allo-Allo spatial judgments compared to Allo-Ego spatial judgments. No further significant comparisons were found (at least *p* < 0.05 Corr_FDR_).

**Table 4:** Channels with a significant increase in both Hb chromophores (HbO, HbR) between spatial conditions

Contrast	Ch	Regions	Right/Left	Hb	*t* value	*p*-value	Cohen’s d
AA > AE	21	Superior frontal gyrus	L	HbO	2.75	< 0.01	0.45

Only significant results after correction are shown

*AA* allo-allo, *AE* allo-ego

#### Ego-Ego vs Allo-Allo

Results of the direct comparisons between Ego-Ego and Allo-Allo spatial judgements are reported in Table 5. For Ego-Ego compared to Allo-Allo spatial judgements, higher concentrations of ΔHbR only were found bilaterally in frontal regions. Specifically, higher ΔHbR concentrations were found in channels 2, 4, 5, 8, 9, 21, 30 located above Superior and Middle Frontal gyri, and Precentral gyrus (at least *p* < 0.05 Corr_FDR_).

**Table 5:** Channels with a significant increase in both Hb chromophores (HbO, HbR) between spatial conditions

Contrast	Ch	Regions	Right/Left	Hb	*t* value	*p*-value	Cohen’s d
EE > AA	2	Superior frontal gyrus	R	HbR	3.30	< 0.01	0.58
	5	Superior frontal gyrus	L	HbR	3.03	< 0.01	0.52
	8	Middle frontal gyrus	R	HbR	2.94	< 0.01	0.50
	21	Superior frontal gyrus	L	HbR	2.88	< 0.01	0.48
	30	Precentral gyrus	R	HbR	2.47	0.01	0.42
	4	Superior frontal gyrus	R	HbR	2.35	0.01	0.39
	9	Middle frontal gyrus	L	HbR	2.30	0.01	0.41
AA > EE	1	Superior frontal gyrus	L	HbO	3.29	< 0.01	0.55
	20	Inferior parietal gyrus	R	HbR	3.14	< 0.01	0.54
	3	Superior frontal gyrus	L	HbO	3.06	< 0.01	0.51
	21	Superior frontal gyrus	L	HbO	2.95	< 0.01	0.49
	31	Precentral gyrus	L	HbO	2.90	< 0.01	0.48
	32	Angular gyrus	R	HbR	2.68	0.01	0.45
	26	Precentral gyrus	R	HbO	2.67	0.01	0.45
	23	Inferior frontal gyrus	L	HbO	2.59	0.01	0.42
	10	Middle frontal gyrus	R	HbO	2.59	0.01	0.44
	38	Superior parietal gyrus	R	HbO	2.59	0.01	0.45
	43	Inferior parietal gyrus	L	HbO	2.50	0.01	0.43
	16	Inferior frontal gyrus	R	HbO	2.17	0.02	0.36
	2	Superior frontal gyrus	R	HbO	2.03	0.02	0.33

Only significant results after correction are reported

*EE* ego-ego, *AA* allo-allo

For the Allo-Allo spatial judgments higher concentrations of both ΔHbO and ΔHbR were found bilaterally in Frontal and Parietal regions. More specifically, the results revealed higher concentrations of ΔHbO in channels 1–3, 10, 16, 21, 23, 26, 31, 38, 43, covering rostro-caudally Superior, Middle, and Inferior Frontal gyri, the Precentral gyrus, Superior and Inferior Parietal gyri. With respect to ΔHbR, higher concentrations were found in channel 20, 32, placed above Inferior Parietal and Angular gyri (at least *p* < 0.05 Corr_FDR_).

#### Ego-Allo vs Allo-Ego

The results of the direct comparisons between Ego-Allo and Allo-Ego spatial judgements are reported in Table 6. The results showed for Ego-Allo spatial judgments higher concentrations of ΔHbO in channels 12, 17 located over the right Superior Temporal gyrus and left Supramarginal gyrus, respectively, and higher concentrations of ΔHbR in channel 33 located over the left Angular gyrus (at least *p* < 0.05 Corr_FDR_).

**Table 6:** Channels with a significant increase in both Hb chromophores (HbO, HbR) between spatial conditions

Contrast	Ch	Regions	Right/Left	Hb	*t* value	*p*-value	Cohen’s d
EA > AE	17	Supramarginal gyrus	L	HbO	2.49	0.01	0.40
	12	Superior temporal gyrus	R	HbO	2.46	0.01	0.41
	33	Angular gyrus	L	HbR	2.31	0.01	0.40
AE > EA	16	Inferior frontal gyrus	R	HbR	3.73	< 0.01	0.63

Only significant results after correction are shown

*EA* ego-allo, *AE* allo-ego

For Allo-Ego spatial judgments, higher concentrations of ΔHbR were found only in channel 16, located over the right Inferior Frontal gyrus (at least *p* < 0.05 Corr_FDR_).

#### EgoEgo vs Allo-Ego

The results of the direct comparisons between Ego-Ego and Allo-Ego spatial judgments are reported in Table 7. For Ego-Ego spatial judgments vs Allo-Ego ones, higher concentrations of ΔHbR were found bilaterally only in Frontal regions, specifically in channels 4 and 29, located respectively above the Superior Frontal gyrus and Precentral Gyrus (at least *p* < 0.05 Corr_FDR_).

**Table 7:** Channels with a significant increase in both Hb chromophores (HbO, HbR) between spatial conditions

Contrast	Ch	Regions	Right/Left	Hb	*t* value	*p*-value	Cohen’s d
EE > AE	29	Precentral gyrus	L	HbR	3.32	< 0.01	0.58
	4	Superior frontal gyrus	R	HbR	3.15	< 0.01	0.53
AE > EE	32	Angular gyrus	R	HbR	3.66	< 0.01	0.61
	38	Superior parietal gyrus	R	HbO	3.15	< 0.01	0.53
	26	Precentral gyrus	R	HbO	3.01	< 0.01	0.51
	7	Middle frontal gyrus	L	HbO	2.78	< 0.01	0.46
	18	Inferior frontal gyrus	R	HbO	2.77	< 0.01	0.48
	1	Superior frontal gyrus	L	HbO	2.75	< 0.01	0.46
	44	Inferior parietal gyrus	R	HbO	2.68	0.01	0.45
	34	Precentral gyrus	R	HbO	2.55	0.01	0.43
	12	Superior temporal gyrus	R	HbR	2.54	0.01	0.42
	2	Superior frontal gyrus	R	HbO	2.50	0.01	0.42
	18	Supramarginal gyrus	R	HbR	2.42	0.01	0.40
	3	Superior frontal gyrus	L	HbO	2.39	0.01	0.41
	16	Inferior frontal gyrus	R	HbO	2.32	0.01	0.40
	5	Superior frontal gyrus	L	HbO	2.31	0.01	0.39
	43	Inferior parietal gyrus	L	HbO	2.24	0.02	0.39
	9	Middle frontal gyrus	L	HbO	2.19	0.02	0.38
	41	Superior parietal gyrus	L	HbO	2.13	0.02	0.35
	10	Middle frontal gyrus	R	HbO	2.11	0.02	0.36
	4	Superior frontal gyrus	R	HbO	2.10	0.02	0.35
	28	Superior frontal gyrus	R	HbO	2.11	0.02	0.39
	11	Superior frontal gyrus	L	HbO	2.09	0.02	0.36
	22	Superior frontal gyrus	R	HbO	2.08	0.02	0.35
	37	Inferior parietal gyrus	L	HbO	1.96	0.03	0.33

Only significant results after correction are shown

*EE* ego-ego, *AE* ello-ego

For Allo-Ego spatial judgments vs Ego-Ego ones, the results showed higher concentrations of ΔHbO bilaterally in Fronto-Parietal regions, and higher concentrations of ΔHbR more right-sided in Temporo-Parietal regions. More specifically, higher concentrations of ΔHbO were found in channels 1, 2, 3, 4, 5, 7, 9–11, 16, 18, 22, 26, 28, 34, 37–38, 41, 43, 44 located rostro-caudally over the Superior, Middle, and Inferior Frontal gyri, Precentral gyrus, and Superior Parietal gyrus (at least *p* < 0.05 Corr_FDR_). For ΔHbR, higher concentrations were found in channels 12, 18, 32 located respectively above the Supramarginal, Superior Temporal and Angular gyri (at least *p* < 0.05 Corr_FDR_).

#### Allo-Allo vs Ego-Allo

The results of the direct comparisons between Allo-Allo and Ego-Allo spatial judgments are reported in Table 8. For Ego-Allo spatial judgments vs Allo-Allo ones, higher concentrations of both ΔHbO and ΔHbR were found bilaterally in Parieto-Temporal and Occipital regions. More specifically, higher concentrations of ΔHbO were found in channels 24, 25, placed respectively above the right-Superior and left-Inferior Parietal gyri (at least *p* < 0.05 Corr_FDR_). For ΔHbR, higher concentrations were found in channels 18, 19, 33, 37, which are located over the right Supramarginal, left Inferior Parietal, left Angular and Superior Occipital gyri (at least *p* < 0.05 Corr_FDR_).

**Table 8:** Channels with a significant increase in both Hb chromophores (HbO, HbR) between spatial conditions

Contrast	Ch	Regions	Right/Left	Hb	t value	p-value	Cohen’s d
EA > AA	25	Inferior parietal gyrus	L	HbO	3.22	< 0.01	0.55
	19	Inferior parietal gyrus	L	HbR	3.18	< 0.01	0.54
	18	Supramarginal gyrus	R	HbR	2.86	< 0.01	0.49
	37	Superior occipital gyrus	L	HbR	2.47	0.01	0.46
	24	Superior temporal gyrus	R	HbO	2.39	0.01	0.39
	33	Angular gyrus	L	HbR	2.30	0.01	0.39

Only significant results after correction are shown

*EA* ego-allo, *AA* allo-allo

### fNIRS Results: Comparisons Between Left and Right Hemisphere

The results of the direct comparisons between channels in the left and right hemispheres in the Ego-Ego, Allo-Allo, Ego-Allo and Allo-Ego conditions are reported in Table 9.

**Table 9:** Channels with a significant increase in both Hb chromophores (HbO, HbR) between left and right hemispheres for each condition

Contrast	Ch	Regions	Condition	Hb	*t* value	*p*-value	Cohen’s d
Left > Right	19/20	Inferior Parietal gyrus	EE	HbR	3.95	0.00	0.67
	25/26	Superior Frontal gyrus	AA	HbR	3.30	0.00	0.59
	29/32	Superior Frontal gyrus	EA	HbO	3.30	0.00	0.58
	7/8	Superior Frontal gyrus	EA	HbO	3.12	0.00	0.54
Right > Left	24/23	Superior Frontal gyrus	EE	HbO	2.34	0.00	0.55
	24/23	Superior Frontal gyrus	AE	HbO	3.45	0.00	0.59

Only significant results after correction are shown

*EE* ego-ego, *AA* allo-allo, *EA* ego-allo, *AE* allo-ego

#### Ego-Ego

The Left vs Right hemisphere contrast revealed an increase of ΔHbR in channel 19, covering the Inferior Frontal gyrus (*p* < 0.01 Corr_FDR_). The Right vs Left hemisphere contrast revealed an increase of ΔHbO in channel 24 covering the Superior frontal gyrus (*p* < 0.01 Corr_FDR_).

#### Allo-Allo

The Left vs Right hemisphere contrast revealed an increase of ΔHbR in channel 25, covering the Superior Frontal gyrus (*p* < 0.01 Corr_FDR_).

#### Ego-Allo

The Left vs Right hemisphere contrast revealed an increase of ΔHbO in channels 7 and 29, covering the left Superior Frontal gyrus (*p* < 0.01 Corr_FDR_).

#### Allo-Ego

The Right vs Left hemisphere contrast revealed an increase of ΔHbO in channel 24 covering the right Superior frontal gyrus (*p* < 0.01 Corr_FDR_).

## Discussion

The aim of the present study was to investigate the cortical correlates of switching and non-switching processes between egocentric and allocentric frames of reference. Participants had to provide switching (from-ego-to-allo; from-allo-to-ego) and non-switching (only-egocentric; only-allocentric) spatial judgments about memorised triads of objects, while task-related hemodynamic responses were measured using the fNIRS neuroimaging technique.

Overall, both behavioural and neurofunctional results showed differences between non-switching and switching spatial judgments.

With regard to the behavioural results, participants were faster and more accurate in providing non-switching egocentric spatial judgments (Ego-Ego) than all other judgments. Instead, participants were slower and less accurate in providing non-switching allocentric judgments (Allo-Allo) than Ego-Allo switching judgments. Finally, in switching conditions, participants were more accurate when the first reference frame was egocentric (Ego-Allo) rather than allocentric (Allo-Ego). In line with previous studies (cf. Ruggiero et al. 2018a b; Orti et al. 2023), this pattern of data demonstrated the facilitation of spatial representations anchored to an egocentric reference system, considered as the primary spatial encoding system for body-environment interactions (e.g., Millar 1994; Iachini and Logie 2003; Milner and Goodale 2006, 2008; Goodale and Milner 2018).

### Neurofunctional Data: Non-Switching Processes

Ego-Ego non-switching spatial judgments were mainly supported by widespread cortical activities in parietal and frontal regions, where a bilateral and selective increase in HbR was found. In particular, the Ego-Ego > baseline and EgoEgo > AlloAllo contrasts revealed increased caudo-rostral cortical activity in the precentral gyrus (channels 29–30) as well as in the superior and middle frontal gyri (channels 2, 4–5, 8–9, 21). The EgoEgo > EgoAllo contrasts showed increased caudo-rostral cortical activity in the superior parietal (channel 5, 7, 10), precentral (channels 4, 9, 16, 23), superior and inferior frontal gyri (channels 2, 8, 27, 29, 31). Finally, the EgoEgo > AlloEgo contrast showed increased cortical activity in the precentral and superior frontal gyri (channels 4, 29). Overall, the involvement of the fronto-parietal regions (i.e., superior parietal, precentral, superior, and middle frontal gyri) in Ego-Ego spatial judgments is largely in line with previous results coming from fMRI studies with perceptual and spatial memory paradigms (see Galati et al. 2000, 2010; Ruotolo et al. 2019; Derbie et al. 2021b). However, while in our study a parietal activation emerged only from the Ego-Ego > Ego-Allo contrast (superior parietal gyrus; channels 5, 7, 10), previous evidence reported a greater activation in the frontal and parietal regions during subject-centred compared to object-centred spatial judgments (Galati et al. 2000; Ruotolo et al. 2019). We can argue that differences can be ascribed to the nature of tasks, stimuli, procedure and manipulation for egocentric and allocentric conditions (see also Committeri et al 2004).

In addition, the analyses revealed greater activations in the left inferior frontal gyrus (channel 19), where an increase in HbR was observed, and in the right superior frontal gyrus (channel 23), with an increase in HbO concentrations. The first could be related to strategic orientation of the attentional focus (Corbetta et al. 2008), the latter seems to be related to the adoption of a view-centered perspective (Committeri et al. 2004).

AlloAllo non-switching spatial judgments were mainly supported caudo-rostrally by inferior-parietal and frontal regions, where bilateral increases in both HbO and HbR were observed. In particular, the AlloAllo > Baseline contrasts revealed an increase in HbO in the superior and middle frontal gyri (channels 1, 7) and an increase in HbR in the precentral gyrus (channel 25). In particular, the AlloAllo > AlloEgo contrast only showed a selective increase in HbO in the superior frontal gyrus (channel 21). Of particular interest is the AlloAllo > EgoEgo contrast, which revealed a caudo-rostral increase in HbO concentrations in the precentral gyrus (channels 26, 31), superior, middle, and inferior frontal gyri (channels 1–3, 10, 16, 21, 23), and an increase in HbR concentrations in the inferior parietal (channels 20, 38, 43) and angular gyri (channel 32). In sum, the involvement of the left superior frontal gyrus along with the other frontal areas may reflect the efforts for the active maintenance in working memory of spatial information (Boisgueheneuc et al. 2006; Barbey et al. 2013; Derbie et al. 2021a, b). Besides, the angular gyrus, along with the frontal regions, would also be involved in the process of detaching from a body-centered perspective to represent an object-centered one (Arzy et al. 2006; Blanke et al. 2005; Derbie et al. 2021a; Gramann et al. 2010; see also Derbie et al. 2021b). This overall increased activity in frontal areas is consistent with the behavioural results discussed above. In fact, participants found allocentric judgments more difficult (i.e., slower and less accurate) than egocentric ones.

Interestingly, the Ego-Ego and Allo-Allo > Baseline contrasts revealed a partial overlap between brain regions subserving egocentric and allocentric spatial processing. Indeed, both contrasts revealed an increased cortical activity in frontal regions, more specifically in superior and middle frontal gyri. Notably, such a common pattern of activations could be related to the maintenance in working memory of the set of rules or the response mapping needed to account for both spatial tasks (Cutini et al. 2008; see also Derbie et al. 2021a, b). Alternatively, the convergence between egocentric and allocentric spatial reference frames across frontal regions could also be explained by the mediation of the dorsal attentional network, which mediates both spatial codings as suggested by Derbie and colleagues (Derbie et al. 2021a, b). This explanation is consistent with previous evidence supporting the idea that the egocentric component is subsumed from the allocentric one (Zaehle et al. 2007).

### Neurofunctional Data: Switching Processes

AlloEgo spatial judgments were mainly supported by frontal regions, as shown by the AlloEgo > Baseline contrast, which revealed higher concentrations of HbO in the superior, middle, and inferior frontal gyri (channels 5, 7, 18). The AlloEgo > EgoAllo contrasts showed greater concentrations of HbR in the inferior frontal gyrus (channel 16). Instead, the AlloEgo > EgoEgo contrast revealed higher caudo-rostral concentrations of HbO in the superior and inferior parietal gyri (channels 37–38, 41, 43–44), precentral gyrus (channels 26, 34) and superior, middle and inferior frontal gyri (channels 1, 3–5, 7, 9–11, 16, 18, 22, 28), as well as in regions close to the temporo-parietal junction such as the superior temporal, angular and supramarginal gyri (channels 12, 18, 32) for HbR. Furthermore, the lateralization analysis revealed a greater activity in the right superior frontal gyrus (channel 24) with an increase of HbO concentrations. In line with previous neuroimaging studies, such activation could reflect the primary process of coordinate (i.e., metric) spatial information (Ruotolo et al. 2019).

Interestingly, Ego-Allo switching spatial judgments were supported by widespread activation involving the caudo-rostral temporal, parietal and frontal regions, where bilateral increases in both HbO and HbR were found. More specifically, the EgoAllo > Baseline contrast revealed that the process of translation from an egocentric to an allocentric reference frame was supported caudo-rostrally by the superior and middle temporal gyri (channels 14, 24), the supramarginal gyrus (channel 17), the superior and inferior parietal gyri (channels 26, 41–42), and finally the superior, middle and inferior frontal gyri (channels 5, 7–9, 16, 18, 20), where a selective increase in HbO was observed. Similarly, the EgoAllo > EgoEgo contrast showed bilaterally higher concentrations of HbO in the superior and inferior parietal gyri (channels 41, 44), the superior, middle, and inferior frontal gyri (channels 3, 7, 9, 16, 23), and increased HbR in regions adjacent to the temporo-parietal junction, the angular and supramarginal gyri (channels 18, 32), superior and inferior parietal gyri (channels 20, 39). The EgoAllo > AlloAllo contrast showed bilaterally higher concentrations of HbO in the superior temporal (channels 24) and inferior parietal gyri (channels 25), and of HbR in the superior occipital gyrus (channels 37), the angular and supramarginal gyri (channels 18, 33) and the inferior parietal gyrus (channels 19). The EgoAllo > AlloEgo contrast showed bilaterally higher concentrations of HbO in the superior temporal (channel 12) and supramarginal gyri (channel 17) and increased HbR in the angular gyrus (channel 33).

Of particular interest is the neurofunctional activity found at the temporo-parietal junction, where a significant increase in HbR was observed in both ego-allo and allo-ego switching conditions. We argue that the set of cortical activity at the temporo-parietal junction plays a central role in the translation processes between spatial representations. In this regard, recent work by Wolff and colleagues (Wolff et al. 2018) has suggested that the modulation of neural processes measured by event-related potentials (ERP) at the temporo-parietal junction underlies the preparatory processes required to switch between response sets. Such neuromodulation, underpinning the preparatory phase prior to switching, is in turn modulated by changes in the noradrenergic pathway from the locus coeruleus to the prefrontal cortex (Aston-Jones and Cohen 2005; Wolff et al. 2018). Our results seem consistent with a crucial involvement of the temporo-parietal junction in the preparation phase before switching from one frame of reference to another. Moreover, since we found an increase in HbR rather than HbO at the temporo-parietal junction in EgoAllo/AlloEgo > EgoEgo contrasts, one could argue that this reflects a decrease in cortical activity. Several studies investigating haemodynamic responses with event-related paradigms combining fMRI and fNIRS have reported a correlation between HbR and BOLD responses (Wobst et al. 2001; MacIntosh et al. 2003; Huppert et al. 2006a; Wijeakumar et al. 2017). A correlation between HbO and BOLD responses has also been reported in studies combining fMRI and fNIRS, where haemodynamic responses were investigated with functional paradigms inducing sustained activations (Yamamoto and Kato 2002; Strangman et al. 2003). Such results seem to reflect the phenomena of “undershoot” and “overshoot” of the BOLD signals related to the decrease and increase of HbR concentrations, respectively. The undershoot of the BOLD signal is related to the early decrease of HbR due to the blood washout effect—and the consequent increase of HbO—at stimulus onset, whereas the overshoot is related to the increase of HbR at stimulus offset due to the cessation of HbO supply (Steinbrink et al. 2006). In the present study, we designed an experimental paradigm lasting 18 s to induce sustained activation. Since a long experimental paradigm evokes higher signal amplitude changes in HbO than in HbR, we should expect higher concentrations of HbO as a marker of underlying task-related neural activity. On the other hand, and based on the above, we argued that higher concentrations of HbR might mask an important but non-sustained task-related neural activity (i.e., an overshoot in the BOLD signal). In line with previous studies, the local increase in HbR at the temporo-parietal junction in the switching conditions could be interpreted as evidence for an early involvement of such brain regions in the process of translation between frames of reference, and more specifically in the “prepare-to-switch” phase. Thus, the increase in HbR concentrations in the Temporo-Parietal Junction under switching conditions might be related to the process of pre-allocation of mental resources (i.e., “adjusting the level of preparedness”, Wolff et al. 2018), which is required to translate spatial information between frames of reference. These results are also consistent with the “third stream hypothesis” proposed by Rizzolatti and Matelli (2003), which suggests a differentiation within the dorsal stream into “dorso-dorsal” and “ventro-dorsal” streams.

Although the results of the present work suggest a central role of the Temporo-Parietal junction in switching processes between spatial representations, it is worth of noting that this brain region represents an important hub in different brain networks serving several higher order cognitive processes (Igelström, and Graziano 2017). Previous fMRI studies have reported foci of neural activity in the Temporo-Parietal Junction with different switching-like paradigms such as the Wisconsin Card Sorting Test (Lie et al. 2006), spatial attention reorientation (Thiel et al. 2004), auditory spatial attention switching (Larson and Lee 2013), rule or stimulus categorization switching (Philipp et al. 2013), switching to alterative resolutions of moral dilemmas (Tei et al. 2017). Taken together, all these studies point out to the Temporo-Parietal Junction as a circuit breaker that aims to select the most appropriate action rule. On the other hand, to the best of our knowledge, the present study is the first to report a significant activation of the Temporo-Parietal Junction subserving visuo-spatial switching processes.

As in the non-switching condition, the involvement of the left superior frontal gyrus (channels 7, 29) revealed by the lateralization analysis could be related to the detachment process from an egocentric-to-allocentric perspective (Iachini and Logie 2003) and/or could be related to the active maintenance of spatial information and response mapping in working memory (Boisgueheneuc et al. 2006; Barbey et al. 2013; Derbie et al. 2021a, b).

At this point it is important to discuss the pattern of neurofunctional data in the light of the results of the behavioral tasks. As reported above, Ego-Allo spatial judgments were more accurate than Allo-Ego ones. Coherently, the Allo-Ego > baseline contrast again showed a spread of activation across frontal regions (i.e., inferior, middle, and superior frontal gyri) compared to the Ego-Allo > baseline contrast (i.e., superior, and middle frontal gyri). This result is noteworthy as it could be interpreted as the inherent difficulty of making switching judgments from an allocentric perspective, as reported in previous studies (Ruggiero et al. 2018a; Orti et al. 2023).

In summary, in line with our hypothesis the translational processes between egocentric and allocentric reference frames appear to be supported rostro-caudally by a fronto-parieto-temporal network. To the best of our knowledge, this is the first study to report the simultaneous activation of the fronto-parietal regions, where body-centred spatial representations are thought to be stored, and of the parieto-temporal regions, where object-centered spatial representations are thought to be stored. Crucially, the temporo-parietal junction appears to play a pivotal role in such translational processes between body-centered and object-centered spatial representations.

However, although the main hypotheses on which the paper is based refer to visuo-spatial processes (Burgess 2006), the results reported above may also reflect modulations of attentional processes. Indeed, the switch between reference systems cannot be assumed to be an exclusively spatial task, since the translation of spatial information between egocentric and allocentric reference systems also requires a shift of attention from a body-centred to an object-centred perspective and vice versa. Such switching processes would lead to an interplay between the dorsal and attentional networks serving egocentric and allocentric spatial processing, respectively (Corbetta and Shulman 2002; Corbetta et al. 2008; Vossel et al. 2014). Consistently, the neurofunctional results confirm the role of the dorsal and ventral attentional networks in the egocentric and allocentric spatial encodings, respectively (Corbetta and Shulman 2002; Corbetta et al. 2008; Kravitz et al. 2013; Vossel et al. 2014; see also Derbie et al. 2021a). The simultaneous activation of these attentional networks in both switching conditions is likely due to the dynamic reorientation processes required to transpose spatial information between egocentric and allocentric reference frames (see Vossel et al. 2014 for a relevant review; see also Corbetta et al. 2008).

Finally, it should be noted that the study presents some limitations. Since the fNIRS technique has a limited depth resolution (~ 1.5 cm below the scalp, Pinti et al. 2019), the cortical activations reported above originate from the superficial layers of the cortex. For this reason, activations in deeper neural structures that are thought to support spatial switching processes (e.g., RSC) were not reported in the present study. Furthermore, although the temporal dependencies of activations between the different brain regions involved in translation processes between reference frames were beyond the scope of this work, future studies could investigate the functional connectivity between the fronto-parieto-temporal regions in translational processes between spatial representations.

## Conclusions

Given the natural complexity of the environment in which we move and act every day, switching between egocentric and allocentric spatial frames of reference is a fundamental capacity. Using fNIRS neuroimaging, we investigated the cortical brain activity underlying the translational (and non-translational) processes of visuo-spatial information in terms of egocentric and allocentric frames of reference.

In the present study we found that this visuospatial process requires the cooperation of two fronto-parietal networks (Galati et al. 2000; Corbetta and Shulman 2002). Furthermore, the role of the brain regions belonging to the Temporo-Parietal Junction in switching between reference frames is noteworthy. Indeed, our results suggest that this junction is involved early in the switching between body- and object-centred frames of reference. This early involvement is probably due to the role of this brain region in the preparation phase, which pre-allocates the cognitive resources required for switching processes.

At the theoretical level, the present results are consistent with the visuospatial memory model (i.e., the “two-system model”) proposed by Burgess and colleagues (Burgess 2006, 2008; Byrne et al. 2007), according to which egocentric and allocentric spatial representations cooperate. Future studies should investigate how typical or pathological neurodegenerative disorders might affect the hemodynamic brain activity associated with switching (and non-switching) between reference frames.

In terms of practical implications, the present study could pave the way for further investigations of fNIRS-related brain activity underlying the switching processes between reference frames in typical or pathological ageing (see Harris et al. 2012; Harris and Wolbers 2014; Ruggiero et al. 2018a).
